# Supplementary material for: A Likelihood Approach for Real-Time Calibration of Stochastic Compartmental Epidemic Models
Source: PLoS Comput Biol. 2017 Jan 17;13(1):e1005257. doi: 10.1371/journal.pcbi.1005257 (PMC5240920; doi:10.1371/journal.pcbi.1005257)
Supplement: S1 File — (TAR.GZ) [file pcbi.1005257.s014.tar.gz › HSPH_Online-SI-Revision/MSS10/n1-extreme/n1-extreme_NEW2_summary2.pdf]

peak -8	3w spec	3w cum	5w spec	5w cum	end	3w spec LOG	3w cum LOG	5w spec LOG	5w cum LOG	end LOG
mean	143.1	131.7	83.7	96.2	30.9	0.6	0.5	0.7	0.5	0.4
median	87.1	80.8	79.3	80.7	28.9	0.6	0.5	0.6	0.5	0.3
quantile .1	48.6	42.4	38.	42.2	12.3	0.2	0.2	0.2	0.2	0.1
quantile .9	261.5	235.6	137.5	143.3	50.1	1.1	0.8	1.3	0.2	0.8
40	39	36	38	32						
peak -4	3w spec	3w cum	5w spec	5w cum	end	3w spec LOG	3w cum LOG	5w spec LOG	5w cum LOG	end LOG
mean	32.3	29.7	35.5	27.9	23.4	0.2	0.2	0.3	0.2	0.2
median	31.1	27.4	32.3	24.8	17.5	0.2	0.1	0.2	0.1	0.1
quantile .1	18.7	16.8	14.3	14.8	5.7	0.1	0.1	0.1	0.1	0.
quantile .9	46.5	44.6	49.2	47.2	41.4	0.4	0.3	0.6	0.1	0.3
38	39	30	28	30						
peak	3w spec	3w cum	5w spec	5w cum	end	3w spec LOG	3w cum LOG	5w spec LOG	5w cum LOG	end LOG
mean	27.7	20.4	35.3	21.5	24.4	0.2	0.1	0.3	0.1	0.1
median	21.5	17.3	28.8	16.7	20.4	0.1	0.1	0.1	0.1	0.1
quantile .1	11.9	6.8	16.3	5.1	6.6	0.1	0.	0.1	0.	0.
quantile .9	54.6	38.5	68.5	45.4	49.5	0.4	0.2	0.6	0.	0.3
33	27	32	26	23						
peak +4	3w spec	3w cum	5w spec	5w cum	end	3w spec LOG	3w cum LOG	5w spec LOG	5w cum LOG	end LOG
mean	49.3	29.4	56.3	31.1	33.9	0.3	0.2	0.4	0.2	0.2
median	37.8	22.3	45.3	26.7	25.1	0.2	0.1	0.2	0.1	0.1
quantile .1	18.2	9.8	24.4	9.2	7.2	0.1	0.	0.1	0.	0.
quantile .9	82.8	52.8	94.2	62.9	76.9	0.6	0.4	0.8	0.	0.5
24	18	34	13	13						
peak +8	3w spec	3w cum	5w spec	5w cum	end	3w spec LOG	3w cum LOG	5w spec LOG	5w cum LOG	end LOG
mean	88.8	52.9	208.6	54.8	57.8	0.4	0.3	0.5	0.3	0.3
median	48.5	46.2	67.4	47.3	53.8	0.2	0.2	0.4	0.2	0.2
quantile .1	26.1	11.6	37.8	13.4	11.9	0.1	0.	0.2	0.1	0.1
quantile .9	127.	92.9	396.	92.1	90.3	0.8	0.5	0.9	0.1	0.6
32	17	34	13	13	{}	{}	{}	{}	{}	{}
